# Supplementary material for: Content-rich biological network constructed by mining PubMed abstracts
Source: BMC Bioinformatics. 2004 Oct 8;5:147. doi: 10.1186/1471-2105-5-147 (PMC528731; doi:10.1186/1471-2105-5-147)
Supplement: Additional File 2 — The original results of the above study (non-essential files are deleted to keep the file size under the limit set by BMC bioinformatics). [file 1471-2105-5-147-S2.bz2 › chilibotAdditionalFile2/dip40/727ID9822594E18076/html/HLYB_HLYA.html]

 


 **HLYB** and **HLYA** 
  
Found 91 abstracts in PubMed, retrieved 40.  
 

 What does Google say? 
 PDF only 
| .edu only 

---

**Interactive relationship** (e.g. stimulation, inhibition, etc)

**Neutral relationship**- This interaction is likely to involve either binding of  **HlyA**  to  **HlyB**  or activation of the transport mechanism.  Ref: 8366127 J Biol Chem, 1993
- A heterologous membrane protein domain fused to the C terminal ATP binding domain of  **HlyB**  can export Escherichia coli hemolysin  [ **HLYA** ] .  Ref: 1400227 J Bacteriol, 1992
- Our data suggest that TnhlyAs generated hemolysin  [ **HLYA** ]  fusion proteins are secreted efficiently by the  **HlyB**  HlyD TolC hemolysin  [ **HLYA** ]  secretion machinery.  Ref: 9711856 FEMS Microbiol Lett, 1998
- We propose that these locations are close to sites on  **HlyB**  that interact with the C terminal signal sequence of  **HlyA** .  Ref: 8366127 J Biol Chem, 1993
- This hybrid protein replaced the NH2 terminal 467 amino acid portion of  **HlyB**  and could still export the Escherichia coli hemolysin  [ **HLYA** ]   **HlyA** .  Ref: 1400227 J Bacteriol, 1992
- Preliminary sequence analysis indicated that another open reading frame homolog to the  **hlyB**  gene is located close to the 3 end of EHEC  **hlyA** .  Ref: 7868227 Infect Immun, 1995
- The secretion of the 107 kDa hemolysin  [ **HLYA** ]  A  **HlyA**  from Escherichia coli is mediated by membrane proteins hemolysin  [ **HLYA** ]  B  **HlyB**  and hemolysin  [ **HLYA** ]  D HlyD.  Ref: 7703231 Biochemistry, 1995
- These lie downstream of  **hlyA**  and  **hlyB** , which encode the El Tor hemolysin  [ **HLYA** ]  and methyl accepting chemotactic factor, respectively.  Ref: 9371455 J Bacteriol, 1997
- The mutant  **HlyB**  HlyDDelta45 inner membrane complex engaged the hemolysin  [ **HLYA** ]  substrate, but this substrate engaged complex failed to trigger recruitment of TolC.  Ref: 11676535 J Mol Biol, 2001
- Point mutants in  **HlyB**  compensate for a deletion in the predicted amphiphilic helix region of the  **HlyA**  signal.  Ref: 7782350 J Biol Chem, 1995
- Identification and preliminary characterization of temperature sensitive mutations affecting  **HlyB** , the translocator required for the secretion of haemolysin  **HlyA**  from Escherichia coli.  Ref: 7808392 Mol Gen Genet, 1994
- Selection for transport competence of C terminal polypeptides derived from Escherichia coli hemolysin  [ **HLYA** ]  the shortest peptide capable of autonomous  **HlyB**  HlyD dependent secretion comprises the C terminal 62 amino acids of  **HlyA** .  Ref: 7531275 Mol Gen Genet, 1994
- We show that an scFv  **HlyA**  hybrid secreted by Hly type I transporter TolC,  **HlyB** , HlyD is accumulated in the extracellular medium with the disulphide bonds correctly formed.  Ref: 11309117 Mol Microbiol, 2001
- These suppressor mutations demonstrated a direct interaction between  **HlyA**  and  **HlyB** .  Ref: 7782350 J Biol Chem, 1995
- This set of  **HlyB**  mutants identifies further sites in  **HlyB**  that modulate substrate specificity but display allele specific effects on a range of  **HlyA**  signal mutants.  Ref: 7782350 J Biol Chem, 1995
- However, we found that  **HlyA**  was present in the periplasm of strains that expressed the TetC  **HlyB**  hybrid.  Ref: 1400227 J Bacteriol, 1992
- Together with the inner membrane protein HlyD and the outer membrane protein TolC,  **HlyB**  is responsible for transport of the 107 kDa toxin  **HlyA**  from the cytoplasm, across both membranes of the cell envelope of Escherichia coli, directly to the medium.  Ref: 8366127 J Biol Chem, 1993
- Mutations in  **hlyB**  affecting secretion could be assigned to two classes those that restore the level of vancomycin resistance to that of E. coli not secreting  **HlyA**  and those that still confer hypersensitivity to the drug in the presence of  **HlyA** .  Ref: 7808392 Mol Gen Genet, 1994
- The system which consists of the two specific membrane proteins  **HlyB**  and HlyD and the outer membrane protein TolC, recognizes on  **HlyA**  a C terminally located signal sequence of about 60 amino acids.  Ref: 9382730 Behring Inst Mitt, 1997
- This article focuses on these bacterial ABC exporters, in particular the  **HlyB**  protein which is central to secretion of the 110 kDa hemolysin  [ **HLYA** ]  across both the cytoplasmic and outer membranes.  Ref: 8453066 Semin Cell Biol, 1993
- The genetic determinants for a fusion of its B subunit StxB, which mediates toxin binding to target cells, with the COOH terminus of Escherichia coli hemolysin  [ **HLYA** ]  A Stx  **HlyA**  has been combined with the determinants of the accessory translocator proteins  **HlyB**  and HlyD in a Notl cassette.  Ref: 8905616 Microb Pathog, 1996
- The export of Escherichia coli hemolysin  [ **HLYA** ]  across the cytoplasmic and the outer membranes requires the COOH terminal signal sequence of  **HlyA** , the two specific translocator proteins  **HlyB**  and HlyD, and the outer membrane protein TolC.  Ref: 9630987 Nat Biotechnol, 1996
- Secretion of Escherichia coli hemolysin  [ **HLYA** ]  is mediated by a sec independent pathway which requires the products of at least three genes,  **hlyB** , hlyD and tolC.  Ref: 7816028 Mol Gen Genet, 1994
- We have isolated transport deficient mutants of  **HlyA**  altered in the C terminal signal sequence and used one of these, a deletion of 29 amino acids, to select compensatory mutants in the transporter protein  **HlyB** .  Ref: 8366127 J Biol Chem, 1993
- We engineered plasmid pETR14, containing the  **hlyB**  and hlyD genes of the Escherichia coli hemolysin  [ **HLYA** ]  operon, to express a fusion protein containing 720 amino acid residues from the nontoxic, receptor binding, carboxy terminus of C. difficile toxin A and the secretion signal of E. coli hemolysin  [ **HLYA** ]  A.  Ref: 9199470 Infect Immun, 1997

**Non-interactive relationship** (e.g. studied together, co-existance, homology, etc.)

- This PagC HlyAs fusion protein was expressed and efficiently secreted into the culture supernatant by E. coli upon complementation with the hemolysin  [ **HLYA** ]  secretion proteins  **HlyB**  and HlyD.  Ref: 8766698 Appl Microbiol Biotechnol, 1996
- An Escherichia coli strain with wild type hemolysin  [ **HLYA** ]  secretion genes  **hlyB**  and hlyD and containing a cloned frpA gene secreted FrpA.  Ref: 8514394 Infect Immun, 1993
- Mutant  **HlyB**  that bound, but did not hydrolyse ATP, supported IM complex assembly, substrate recruitment and bridging, but  **HlyA**  stalled in the channel.  Ref: 9822594 EMBO J, 1998
- In the present study, we selected  **HlyB**  alleles that complemented an internal deletion of 29 amino acids in  **HlyA**  containing a predicted amphiphilic helix region immediately upstream from the previous deletion.  Ref: 7782350 J Biol Chem, 1995
- Our data suggest that all beta Gal HlyAs fusion proteins generated are secreted, albeit with varying efficiencies, by the  **HlyB**  HlyD TolC hemolysin  [ **HLYA** ]  secretion machinery under Sec proficient conditions.  Ref: 8842146 Mol Gen Genet, 1996
- However, hemolytic activity was found in the broth culture supernatant after transforming EDL 933 with the recombinant plasmid pRSC6 carrying the  **hlyB**  and hlyD genes from the E. coli alpha hemolysin  [ **HLYA** ]  operon.  Ref: 7868227 Infect Immun, 1995
- However, HasA is secreted neither by the PrtD PrtE PrtF transporter the genuine E. chrysanthemi protease transporter nor by the  **HlyB**  HlhD TolC transporter the hemolysin  [ **HLYA** ]  transporter.  Ref: 8071214 J Bacteriol, 1994
- The deduced amino acid sequence showed significant homology to the C terminal region of  **HlyB**  31.1% identical residues, a protein located in the Escherichia coli alpha hemolysin  [ **HLYA** ]  operon that is a representative bacterial ATP binding cassette export protein.  Ref: 9401046 J Bacteriol, 1997
- Hemolysin  [ **HLYA** ]  B  **HlyB**  is a membrane bound transport protein composed of an amino terminal multiple membrane spanning portion followed by a conserved ATP binding sequence.  Ref: 8366127 J Biol Chem, 1993
- This recombinant plasmid expressed a fusion protein Gcp F which was secreted into the culture supernatant by E. coli cells when the alpha hemolysin  [ **HLYA** ]  secretion functions  **HlyB**  and HlyD are supplied in trans.  Ref: 8150268 FEMS Microbiol Lett, 1994
- The presence of fragments of genomes  **hlyA** ,  **hlyB** , papAH, papC, sfaG, sfaA and kps MT, associated with the pathogenicity islands of Escherichia coli, in clinical strains of other genera of the family Enterobacteriaceae, has been experimentally evaluated with the use of PCR.  Ref: 12506618 Zh Mikrobiol Epidemiol Immunobiol< MedlineTA>Zh Mikrobiol Epidemiol Immunobiol, 1994
- The fact that point mutations are able to compensate for drastic changes in the signal sequence of  **HlyA**  suggests that substrate specificity of transporters such as  **HlyB**  may shift dramatically during evolutionary history.  Ref: 8366127 J Biol Chem, 1993
- Previously, it was shown that point mutants in the transmembrane domain of the transporter  **HlyB**  could partially correct the transport defect caused by a deletion of the C terminal 29 amino acids of  **HlyA** .  Ref: 7782350 J Biol Chem, 1995
- We have used a mutational approach to investigate a postulated interaction between  **HlyA**  and  **HlyB** .  Ref: 8366127 J Biol Chem, 1993
- The structure and function of the components of the  **HlyA**  secretion apparatus,  **HlyB** , HlyD and TolC, have been studied in great detail.  Ref: 11755084 Trends Microbiol, 2002
- These effects are only observed in the presence of functional  **HlyB**  and TolC, the two other components of the hemolysin  [ **HLYA** ]  secretion machinery.  Ref: 9393456 Mol Gen Genet, 1997
- Extracellular secretion of  **HlyA**  by the TetC  **HlyB**  hybrid required HlyD and TolC.  Ref: 1400227 J Bacteriol, 1992
- We found the CVD 419 probe to be identical to the 3.4 kb HindIII fragment of plasmid pEO40 carrying most of the EHEC  **hlyA**  gene and a part of the putative EHEC  **hlyB**  gene.  Ref: 7868227 Infect Immun, 1995
- Taking these results together, we propose a model for an early stage of initiation of secretion in vivo, in which the NBD of  **HlyB** , specifically recognizes the C terminus of the transport substrate,  **HlyA** , and where secretion is initiated by subsequent displacement of  **HlyA**  from  **HlyB**  by ATP.  Ref: 12662939 J Mol Biol, 2003
- To this end, Salmonella typhimurium SL7207 was used as carrier for secreted p60  **HlyA**  fusion protein by Escherichia coli  **HlyB**  and HlyD transport proteins.  Ref: 7729919 Infect Immun, 1995
- We have purified the nucleotide binding domain of  **HlyB**   **HlyB**  NBD and a C terminal 23kDa fragment of  **HlyA**  plus the His tag HlyA1, which contains the secretion sequence.  Ref: 12662939 J Mol Biol, 2003
- We have developed an export cloning system that is composed of two vectors one in which the fusion of the desired gene with the 3 end of  **hlyA**  is generated, and a second in which the sequences containing the fusion are combined with the accessory genes  **hlyB**  and hlyD, thereby reconstructing the natural organization of the hly locus.  Ref: 9630987 Nat Biotechnol, 1996
- The hydrophobic rich NH2 terminal 34 amino acids of a tetracycline resistance determinant TetC were fused to the COOH terminal 240 amino acids of the hemolysin  [ **HLYA** ]  transporter,  **HlyB** , which contains a putative ATP binding domain.  Ref: 1400227 J Bacteriol, 1992
- The nisT open reading frame is homologous to proteins of the  **HlyB**  hemolysin  [ **HLYA** ]  B protein of Escherichia coli subfamily.  Ref: 1482192 Appl Environ Microbiol, 1992
- This 70 amino acid peptide contains little primary sequence similarity to the  **HlyA**  signal sequence, and we have hypothesized that these signal sequences assume a similar higher order structure which is recognized by the  **HlyB**  D transporter.  Ref: 7703231 Biochemistry, 1995
- The toxin  **HlyA**  is exported from Escherichia coli, without a periplasmic intermediate, by a type I system comprising an energized inner membrane IM translocase of two proteins, HlyD and the traffic ATPase  **HlyB** , and the outer membrane OM porin like TolC.  Ref: 9822594 EMBO J, 1998
- Similar structural homology is observed between these proteins, hemolysin  [ **HLYA** ]  B  **HLYB**  and the major histocompatibility linked peptide transporter, HAM1, the latter two which contain a single MA domain composed of six TM segments.  Ref: 7691068 J Protein Chem, 1993
- Despite the Ts character of the Gly10 substitution, a derivative of  **HlyB** , in which the first 25 amino acids were replaced by 21 amino acids of the lambda Cro protein, was still active in secretion of  **HlyA** .  Ref: 7808392 Mol Gen Genet, 1994
- Bacterial signal peptide independent protein export  **HlyB**  directed secretion of hemolysin  [ **HLYA** ] .  Ref: 8453066 Semin Cell Biol, 1993
